# Traumatic Brain Injury and Artificial Intelligence: Shaping the Future of Neurorehabilitation—A Review

**DOI:** 10.3390/life15030424

**Published:** 2025-03-07

**Authors:** Seun Orenuga, Philip Jordache, Daniel Mirzai, Tyler Monteros, Ernesto Gonzalez, Ahmed Madkoor, Rahim Hirani, Raj K. Tiwari, Mill Etienne

**Affiliations:** 1 School of Medicine, New York Medical College, Valhalla, NY 10595, USArhirani2@student.nymc.edu (R.H.); 2 Department of Psychiatry, Mayo Clinic, Phoenix, AZ 85054, USA; 3 Graduate School of Biomedical Sciences, New York Medical College, Valhalla, NY 10595, USA; 4 Department of Neurology, New York Medical College, Valhalla, NY 10595, USA

**Keywords:** traumatic brain injury, artificial intelligence, rehabilitation

## Abstract

Traumatic brain injury (TBI) is a leading cause of disability and death globally, presenting significant challenges for diagnosis, prognosis, and treatment. As healthcare technology advances, artificial intelligence (AI) has emerged as a promising tool in enhancing TBI rehabilitation outcomes. This literature review explores the current and potential applications of AI in TBI management, focusing on AI’s role in diagnostic tools, neuroimaging, prognostic modeling, and rehabilitation programs. AI-driven algorithms have demonstrated high accuracy in predicting mortality, functional outcomes, and personalized rehabilitation strategies based on patient data. AI models have been developed to predict in-hospital mortality of TBI patients up to an accuracy of 95.6%. Furthermore, AI enhances neuroimaging by detecting subtle abnormalities that may be missed by human radiologists, expediting diagnosis and treatment decisions. Despite these advances, ethical considerations, including biases in AI algorithms and data generalizability, pose challenges that must be addressed to optimize AI’s implementation in clinical settings. This review highlights key clinical trials and future research directions, emphasizing AI’s transformative potential in improving patient care, rehabilitation, and long-term outcomes for TBI patients.

## 1. Introduction

Traumatic brain injury (TBI) is a complex condition with a significant global health burden, characterized by a spectrum of clinical presentations and long-term sequelae. TBI has the highest incidence of all common neurological disorders and is increasingly shown as both an acute and chronic healthcare issue. Approximately 50 million–60 million people around the world have a TBI each year, costing the global economy around USD 400 billion annually [1]. Rehabilitation remains an essential component in the management of TBI, yet current modalities often face limitations in accessibility, accuracy, and personalization. Artificial intelligence (AI) offers transformative potential in addressing these challenges by enhancing diagnostic precision, predicting patient outcomes, and personalizing rehabilitation interventions. AI can have transformative potential as a support system for clinical decision-making with use cases in diagnosis, prognosis, treatment recommendations, and assisted clinical documentation [2]. This review synthesizes recent advancements in AI applications across TBI rehabilitation, encompassing diagnostic tools such as deep learning algorithms for neuroimaging, predictive models for functional and mortality outcomes, and innovative rehabilitative technologies like AI-driven exoskeletons and virtual reality systems. Ethical considerations in AI deployment, including equity, dataset diversity, privacy, and transparency, are also critically discussed. The integration of AI with conventional therapeutic approaches signifies a paradigm shift toward precision rehabilitation, promising improved efficiency and patient-centered care [3]. However, further research is essential to validate these technologies across diverse populations and establish standardized frameworks for clinical implementation. This review is organized into sections addressing the history, applications, and long-term considerations of using AI technology in the rehabilitation of TBI patients.

### Search Strategy

This review examines the role of AI in the promising role of improving TBI rehabilitation outcomes. We consider the challenges and opportunities in the care of TBI patients and the implementation of AI. Our aim is to achieve the following: (i) highlight the considerations of managing TBI; (ii) acknowledge the history and applications of AI within the medical field; (iii) assess the effectiveness of AI as a diagnostic tool for TBI and as a predictor of TBI health outcomes; (iv) gain some insight into clinical trials of AI used in the study of TBI; and (v) create an outlook for long-term considerations for TBI patients. This review synthesizes information from 139 articles identified through a comprehensive literature search in PubMed, Medline, Embase, Web of Science, and the National Institute of Health ClinicalTrials.gov. We included peer-reviewed journal articles, review articles, systematic reviews, and meta-analyses. We only focused on publications written in English. We included articles focusing on human subjects diagnosed with TBI and the use of AI technologies in the rehabilitation of TBI patients. Articles reporting on the effectiveness or outcomes of AI-based interventions in TBI rehabilitation and how AI can be applied in various aspects of rehabilitation were also prioritized.

## 2. Considerations of Managing TBI: Current and Future Perspectives

“Traumatic brain injury” (TBI) refers to a head or body injury that interferes with normal brain function and is the result of an external force or penetrating injury [4]. TBIs can be categorized as mild, moderate, or severe [4]. These categories correspond to the Glasgow Coma Scale (GCS scores) of 13–15, 9–12, and 3–8, respectively [1]. Falls are the most common cause of TBIs, while motor vehicle accidents represent the most common cause of TBI-related death [5]. It has been estimated that 69 million people have a TBI each year globally [6]. Further, TBIs have accounted for the deaths of over 1 million Americans since the start of the twenty-first century [7].

TBIs can lead to a wide range of clinical presentations. Patients can present with disorders of consciousness, conditions which include a minimally conscious state, vegetative state, and coma [5]. They could also present with cognitive dysfunction, conditions which include impaired communication, concentration, memory, attention, processing speed, and executive function [5]. The total cost of TBIs in the United States is estimated to range from USD 60.4–221 billion per year [8]. This significant economic burden, coupled with the considerable impact on patients’ lives, underscores the importance of timely and effective treatment.

Mild TBI (mTBI) has been shown to account for between 58 and 88% of all TBIs [9]. The terms mTBI and concussion are also commonly used interchangeably [9]. According to the American Academy of Neurology, mild TBIs are defined as injuries that involve memory loss or disorientation lasting less than 24 h, and/or loss of consciousness lasting 30 min or less [10].

Vestibular dysfunction, and in particular benign paroxysmal vertigo (BPPV), is commonly observed with mTBI, particularly when post-concussive syndrome is present [11]. BPPV has been shown to be effectively treated using canalith repositioning therapy, and other types of vestibular dysfunction can be treated with vestibular rehabilitation, including gaze stability exercises and gait and balance training, in addition to general reconditioning [11]. Fatigue, another symptom that frequently presents following an mTBI, can be treated using non-specific rehabilitation, such as exercise or specialized interdisciplinary rehabilitation incorporating neuropsychological care, exercise therapy, therapeutic education, and physiotherapeutic coaching [11]. Headache is another common symptom after mTBI. There is a wide range of effective treatments for headache prophylaxis, including oral agents, self-injectable medications, intravenous infusions, trigger point injections, and botulinum toxin injections [12,13]. Likewise, there is a wide range of abortive medications for post-traumatic headaches, including oral medications, nasal sprays, and self-injectable medications. Dysosmia and dysgeusia are also commonly observed symptoms in mTBI patients with post-concussive syndrome, but consistently effective treatments have not been identified for them [11].

Cognitive mTBI symptoms can be treated using cognitive and occupational therapy, but some patients may benefit from brain stimulation procedures such as high-frequency repetitive transcranial magnetic stimulation (rTMS) [11,14,15]. Red/near-infrared light-emitting diodes have also been shown to help alleviate cognitive mTBI symptoms in patients with post-concussive syndrome [11]. In general, cognitive rehabilitation techniques that specifically target individual neuropsychological impairments have shown considerable efficacy when used to treat mTBI patients experiencing post-concussive syndrome [11]. Other techniques that have been helpful include music therapy, art therapy, meditation, and neurofeedback [11].

Psychological symptoms, and in particular anxiety and mood disorders, are also common following mTBIs [11]. These often cause problems sleeping, and can be treated with near-infrared light or pharmacological approaches, the latter of which should be adapted to the specific sleep disorder present [11]. Additionally, animal-assisted therapy, meditation, music therapy, art therapy, and other complementary medicines have also been found to be beneficial after mTBI [16]. Cognitive and Behavioral Therapy (CBT) has also been shown to help with facilitating coping and decreasing anxiety, anger, depression, and PTSD symptoms in TBI patients [11]. Mindfulness-based stress reduction therapy, multidisciplinary care including cognitive rehabilitation, vestibular intervention, headache management, and integrated behavioral care have also all been shown to be beneficial in treating PTSD in mTBI patients [11]. Overall, starting treatment early has been shown to be key in preventing symptoms from becoming chronic, as well as in improving quality of life [11]. Furthermore, it has also been shown that the amount of effort patients exert in therapy sessions is an important predictor of rehabilitation outcomes [17]. Additionally, time spent in more complex therapy activities and the use of specific medications, notably analgesics, have been shown to correlate with improved patient outcomes [17]. Studies have also been conducted investigating the effects of TBIs on activities of daily living. TBI patients show improvements in mobility and independence in daily living but still require support for cognitive, communication, behavioral, and emotional functions, even after 10 years [18]. It has been found that TBIs do not, in and of themselves, increase the risk for adverse driving outcomes such as moving violations or crashes [19].

In recent years, artificial intelligence (AI) has grown in use in TBI treatment. Neurointensive Care Units (NICUs) currently generate extensive amounts of data due to monitoring both neurological imaging results and such physiological parameters as brain tissue oxygenation, cerebral blood flow, cerebral perfusion pressure, intracranial pressure, and others, in addition to vital signs [20]. The Clinical decision support system (CDSS) utilizes AI to analyze patients’ health conditions and predict future events [21].

In a prior study, Hale et al. constructed an artificial neural network (ANN) algorithm with a sensitivity of 99.73% with 98.19% precision, 97.98% accuracy, 91.23% negative predictive value, 0.0027% false negative rate, 60.47% specificity, and AUC = 0.9907 to utilize clinical and imaging data to predict clinically relevant TBIs (CRTBIs) in children [22]. Tu et al. created a predictive model utilizing six machine learning algorithms to assess the risk of mortality for adult TBI patients in the emergency rooms at a Taiwanese medical group system. It made use of such variables as age, sex, and BMI score in addition to clinical findings. Their best predictive model was a logistic regression-based model with an 89.3% accuracy for mortality risk prediction with a sensitivity of 81.2%, specificity of 89.4%, and AUC = 0.925 [23]. In a review of multiple machine learning algorithms, Khalili et al. found that the condition of pupils, the condition of cisterns, and patient age are the most effective predictors of in-hospital mortality for TBI patients, while the condition of pupils, Glasgow Coma Scale motor (GCSm), and patient age are the best predictors of long-term mortality [24]. Further, Pease et al. devised a deep learning fusion model that combined head CT and clinical information (AUC, 0.92 [95% CI: 0.86, 0.97]; *p* < 0.001) that outperformed other compared models to generate 6-month prognoses for patients with severe TBIs. It was found that quantitative CT image analysis helps improve outcome prediction [25]. Finally, Raj et al. presented the first dynamic prognostic algorithm for real-time outcome prediction of patients with TBI treated in the ICU [26]. The use of AI in the NICU to treat TBIs and other neurological conditions has become increasingly sophisticated in recent years and has also reflected the increasing role of AI in medicine at large.

It is important to note that TBI is a very heterogeneous condition. Patients vary widely in terms of injury mechanisms, severity, and outcomes. This poses significant challenges for the development of AI models. TBI can result from different mechanisms such as falls, motor vehicle accidents, or assaults, which can lead to different injury patterns. Models trained on datasets dominated by one injury type may not generalize well to others [27]. TBI severity ranges from mild to severe, with each category potentially requiring different predictive factors. Models developed using data from only severe TBI cases may perform poorly when applied to mild TBI patients [24]. Clinical outcomes from TBI are highly variable, ranging from full recovery to severe disability or death. Outcome variability makes it challenging to develop AI models that accurately predict outcomes across the complete spectrum of TBI severity [28]. TBI datasets used for developing AI models face limitations and biases that can impact the external validity and generalizability of these models.

TBI datasets are often derived from single centers or a limited number of institutions. This can over-represent certain demographics or injury types based on the local population, thus limiting a model’s applicability to diverse populations [29]. Mild TBI cases are often underrepresented in TBI datasets. Many studies elect to focus on moderate to severe TBI, as these patients are more likely to be hospitalized and tracked for follow-up examination. This can lead to models that perform poorly when applied to cases of mild TBI [24]. Conventional outcome measures used in studies on severe TBI may not be sensitive enough to capture the subtle changes present in cases of mild TBI.

In this paper, we will present AI contributions to TBI care. We will explore the origins of AI in healthcare, highlighting case examples that illustrate its effective application in managing TBI. This will include a review of numerous clinical trials involving AI and TBI. The future directions of this technology are promising, and we will highlight the long-term considerations regarding AI use in TBI management.

## 3. Artificial Intelligence in Healthcare: Its History and Applications Within the Field of Neurology

Artificial intelligence (AI) has made significant impacts on multiple industries and its rapid advancements have the potential to revolutionize society, but what is AI and how did this concept come to be? AI is a broad, complex term, that refers to the programming of computers such that they can learn and perform intelligent behavior with minimal human intervention [30]. The roots of AI trace back to the mid-20th century with figures like Alan Turing and John McCarthy and events like the Dartmouth Conference in 1956. Alan Turing’s 1950s paper titled “Computing Machinery and Intelligence” questioned whether man-made machines could execute tasks and solve problems just like humans [31]. This new way of thinking encouraged new discussions among scholars about whether computers could possess intelligence and reasoning similar to humans. Despite Turing posing this question in the year 1950, scholars in this field attribute the origin of AI to the Dartmouth Conference in 1956 and to one of its leading participants, John McCarthy. John McCarthy, who coined the term “artificial intelligence”, envisioned a future where machines could engage in thinking processes and possess abilities, for learning and reasoning allowing them to solve problems like humans or better [32]. After pioneers of the field in the 1950s first introduced the concept of AI, there has been exponential growth in its applications in society.

One industry for which AI’s impact is substantial and promises to bring about major changes is medicine. The exploration of AI in the field of medicine began to advance in the 1970s and 1980s. An important system that emerged during this period was INTERNIST-1 which came into being in 1971. This initial artificial medical advisor employed algorithms to identify illnesses by assessing symptoms. The system did not lighten the load on doctors when it came to diagnosing but served as a helpful resource for verifying their diagnoses [33]. INTERNIST-I performed best when only a single disease was presented in the patient but struggled with complex cases where more than one disease was present. Another development during the same time as INTERNIST-1 was MYCIN. The MYCIN program analyzed patient data including patient history and utilized it to assist doctors in providing the correct antibiotic in the case of an infection. This demonstrated the ability of AI systems to focus on a targeted treatment like appropriate antibiotic administration [34]. MYCIN was never actually used in clinical practice. Observers of the technology raised ethical and legal issues related to the use of computers in medicine and the responsibility of physicians in the event of an incorrect diagnosis. It was also an intensive task to use MYCIN because the user had to enter all the relevant patient information in responses to the MYCIN-prompted questions [35]. Another system called Dxplain was introduced in the 1980s as a tool that enabled healthcare providers to input symptoms for diagnoses; however, Dxpain had a broader range of diagnostic options than INTERNIST-1, making it a more comprehensive tool for physicians working towards their diagnosis [36]. However, it ran into similar issues in implementation as there was a lack of support by clinicians in real-world settings.

The beginning of the 2000s saw the dawn of an era, for AI showcasing its capabilities in different areas like medicine and beyond to a level like none before. IBMs Watson emerged in 2007 as a system of gathering and processing data from various sources. An achievement famously showcased when it triumphed on the game show Jeopardy against human competitors [37,38]. This victory underscored the applications of technology, in the realm of healthcare. In 2017, Watson was used in the field of medicine to pinpoint RNA proteins linked to amyotrophic lateral sclerosis (ALS), showcasing how AI cannot only aid in medical diagnosis and treatment but also in research [39]. AI’s influence is also noticeable in the realm of imaging. Computer Aided Detection (CAD) systems have supported physicians since the 1970s in identifying irregularities in images helping pinpoint a diagnosis [40]. Over the years, advancements in algorithms have allowed them to detect pathologic findings in ultrasound and MRI scans with a level of precision comparable to that of doctors [41]. Dermatology has also seen progress thanks to AI; for instance, an AI system trained on a database of over 120,000 images was able to diagnose skin conditions as accurately as experienced dermatologists [42,43].

The possibilities offered by AI in the field of medicine are extensive and far-reaching. In addition to its applications in radiology and dermatology, the latest studies delve into how AI could anticipate the likelihood of diseases, enhance triage procedures, perfect genetic engineering techniques and even transform medical training by offering inventive methods and materials to prepare future healthcare practitioners [44,45].

AI has a great deal of potential in the field of Neurology, especially when it comes to using neuroimaging. It can impact how we diagnose and treat brain-related conditions more accurately. For example, AI tools are helpful for looking at brain images to recognize and classify types of strokes which can help doctors make treatment decisions faster and, with precision. In cases of multiple sclerosis (MS), AI can be used to forecast how the disease will progress and to categorize patients based on their history and imaging tests which can assist in developing personalized treatment plans. Furthermore, AI has been making advancements in the detection of Alzheimer’s disease by examining brain scans and clinical information to distinguish it from other types of dementia. AI also plays a crucial role in epilepsy by improving the identification and localization of the foci of seizures in the brain through advanced analysis of EEG and MRI data. Although there are still obstacles to be surmounted in utilizing AI in Neurology, AI’s ability to handle and analyze volumes of data, especially imaging, could transform the neurological field [46].

One potential condition for which AI can be helpful within Neurology is in diagnosing and treating TBIs. Advances in AI technology have shown promise in improving the detection and prioritization of findings in head CT scans for TBI treatment and care. A study conducted by Chikamkurthy and colleagues in 2018 highlights how advanced AI models can effectively detect types of intracranial hemorrhages and skull fractures with high accuracy. The study covered a dataset combining 313,318 head CT scans with their respective clinical reports from around 20 clinical centers in India. The algorithms achieved an AUC as high as 0.94 (95% CI 0.92–0.97) for detecting intracranial hemorrhage on certain datasets. In this way, these programs can streamline the assessment process and guarantee prompt attention to a patient with a serious head injury [47]. Likewise, Arbabshirani and colleagues in 2018 highlight the potential for AI to enhance radiology procedures by adjusting the priority of CT scans and expediting the evaluation of situations, like intracranial bleeding. By adopting this method, the time taken for diagnosis, especially in serious cases, is shortened and patient results are improved [48].

The use of AI as a diagnostic tool for TBI has a great deal of promise. The progress in this field showcases how AI has the potential to reshape TBI diagnosis by improving accuracy to boost clinical decision-making. This increases the likelihood of achieving timely, impactful outcomes for patients. The research in this space needs to encompass more prospective trials and varied demographics to ensure that we can limit sample bias and gather comprehensive information in real time.

## 4. The Use of AI as a Diagnostic Tool for TBI

TBIs are estimated to affect 69 million individuals worldwide and are also the leading cause of disability and death for all traumatic insults. It is estimated that mild TBI accounted for 81.02% of injuries, moderate TBI for 11.04%, and severe TBI for 7.95% [49]. The diagnosis and assessment of TBIs are critically important for positive patient outcomes and improved quality of care. A decrease in time to diagnosis is important for timely intervention in severe emergent TBI cases. According to NIH guidelines on the assessment of TBI severity, there are many methods that can aid a clinician in diagnosing TBI. These methods include the use of neuroimaging, determination of altered or loss of consciousness, evaluating posttraumatic amnesia, testing blood-based biomarkers, and the use of criteria such as the Glasgow Coma Scale score [50]. Despite these diagnostic measures, there are still many challenges in the diagnosis of TBIs, such as the absence of clear positive findings on neuroimaging in many cases involving either diffuse or mild injuries [51]. Often in cases of head trauma, swiftness in diagnosis is vital to the administration of the correct treatment and ultimately improving patient outcomes.

Neuroimaging is often the initial modality used by clinicians to diagnose and assess patients with a suspected TBI. Although most patients with an acute TBI do not require neuroimaging, computed tomography (CT) is considered the preferred imaging tool for acute TBIs as it can efficiently and effectively detect structural damage or hemorrhages [52]. However, there are still many challenges in the diagnosis of TBIs due to its heterogeneity and the possible variety of symptom presentation [53]. Current guidelines lack uniformity and standardized assessment methods [54]. Mild TBIs (mTBIs) may not present with symptoms for days to weeks following a traumatic event. Neuroimaging, such as CT could assess overall pathoanatomical findings, yet do not detect minor structural damage [55].

**Figure 1:**
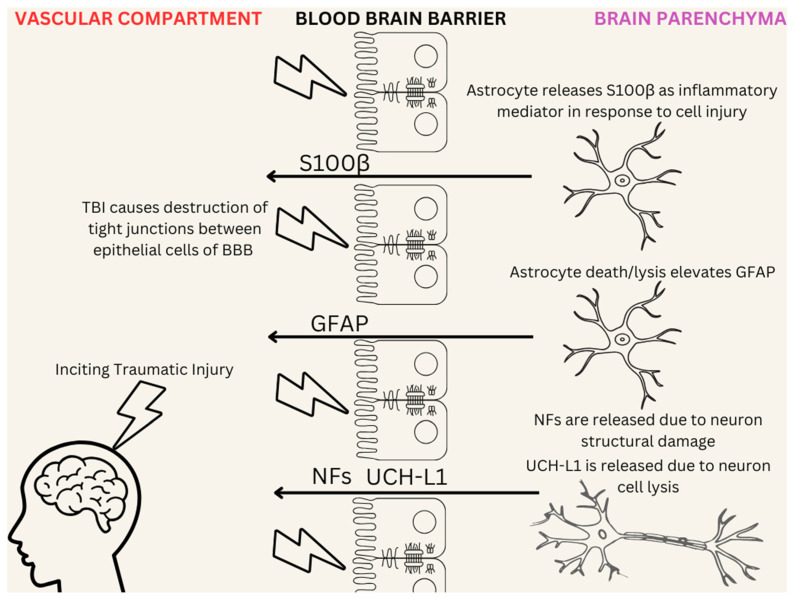
Traumatic brain injury (TBI) can be assessed using several surrogate biomarkers that are released into the bloodstream following injury. Surrogate biomarkers include S100 calcium-binding protein B (S100β), Ubiquitin carboxy-terminal hydrolase L1 (UCH-L1), neurofilaments (NFs), and Glial fibrillary acidic protein (GFAP). These can be detected in the blood through various mechanisms, particularly when the blood–brain barrier (BBB) is disrupted due to injury. When the tight junctions of the BBB are disrupted, proteins may freely enter the vascular compartment.

The necessity for an objective and accurate methodology for diagnosing TBIs has led to the use of blood-based protein biomarkers. These include S100β, Ubiquitin C-Terminal Hydrolase-L1 (UCH-L1), Neurofilament proteins (NFs), and Glial Fibrillary Acidic Protein (GFAP) [56,57]. These biomarkers, also referred to as surrogate markers of TBIs as they are detected within the peripheral blood, can provide a quantifiable way in which TBIs may be diagnosed, without subjecting the patient to ionizing radiation as a result of CT [58]. A systematic review demonstrated that the pattern of biomarker elevation in patients with mTBI is distinct from that observed in severe TBIs [59]. Figure 1 illustrates the role of artificial intelligence in neurorehabilitation for traumatic brain injury, highlighting key applications in diagnostics, treatment planning, and patient monitoring.

Another advantage biomarkers may have over neuroimaging is the ability to differentiate between acute and chronic phases of TBIs, indicated by the elevation of a specific protein [56]. The way in which these biomarkers are quantifiable may yield more significant results for deep learning models as compared to neuroimaging.

However, there are disadvantages to this methodology. Elevated protein Surrogate peripheral biomarkers may only appear as a direct consequence of blood–brain barrier damage (BBBD), as indicated by the 2004 study conducted by Marchi et al. [60]. Not all TBIs may present with BBBD, which limits the utility of such a test. However, a positive test has a high specificity for BBBD. To our knowledge, there is no extensive research conducted regarding the use of deep learning models to assess, quantify, or classify TBIs based on protein biomarkers, although it remains a possible avenue for future discussion.

The use of AI in the context of neuroimaging may be advantageous by allowing diagnoses, and therefore subsequent treatment, to be made more quickly. Deep learning algorithms can assess CT images in an objective and quantitative manner that can supplement the experience and judgment of a clinician. The role of AI in neuroimaging concerning clinical relevance must be determined by supporting data. The data must show significance in favor of its implementation via higher sensitivity, consistency in results, and greater efficiency. The results must also reflect the patient population [61].

In a 2018 review by Chilamkurthy et al., they discussed the accuracy of a deep learning algorithm on the detection and correct assessment of various types of hemorrhages from radiological reports across clinical centers in India, including both in-hospital and outpatient centers. The algorithm achieved a sensitivity of 0.9807 (95% CI 0.9513–0.9947) and specificity of 0.9873 (95% CI 0.9804–0.9922) on its best subset on assessment of hemorrhages. The researchers determined that the difference in sensitivity between the radiologists and the algorithm was not significant (*p* > 0.05), but the algorithm’s specificity was significantly lower (*p* < 0.0001) [47].

Another AI methodology that is associated with TBI neuroimaging is a Convolutional Neural Network (CNN). This network interprets images and segments layers based on characteristics within imaging, improving pattern recognition through large data sets of similar images [62]. CNNs have demonstrated efficacy in the diagnosis of sequelae of TBIs, such as the detection of cerebral microbleeds at a similar accuracy to an experienced clinician [63].

Hematomas following TBI are particularly challenging to diagnose via CT, due to complex factors such as the shape, location, and volume of the hematoma [64,65]. These variables can contribute to errors in neuroimaging analysis. Subtle injuries or microbleeds can be missed by CNNs due to their underrepresentation in the typical dataset. There is, however, potential for improving both the efficiency and accuracy of diagnosis through the use of CNNs.

A current complication with CNNs is that they are highly reliant on large datasets, and many datasets contain more CT imaging than is useful for a deep learning model to interpret for TBI diagnosis. To accommodate for this, 2-D montage images are utilized to avoid a tedious manual selection for the model to learn from. New methods, such as deep montage-based image retrieval (dMIR), allow for a model to classify 2-D to process 3-D images, which are superior for maintaining the context between slices and preserving spatial relationships. A model proposed by Kerley et al. showed an accuracy of 0.988 and a precision of 0.962 out of 1000 heterogenous CTs, with only 12 misclassifications [66].

A 2024 study by Agrawal et al. developed a 3-D CNN model assessing midline shifts (MLS) with CT in TBI patients. The algorithm demonstrated accuracy in measuring MLS up to 30 mm with a mean difference of 0.23 ± 0.52 mm compared to manual measurements in 78 cases. The model was correct in assessing 7/10 positive MLS cases and 4/10 negative MLS cases, with an accuracy of 55%. The specificity was high at 70%, and the sensitivity was moderate at 40% [67]. The researchers noted that the model was only trained on 156 CT scans, and errors may be in part due to the model’s reliance on symmetry, which may vary in normal anatomy between patients. Limitations with ventricular recognition, the key to assessing the extent of a midline shift, may also play a role. However, other deep learning models have been developed to aid in ventricular recognition, by performing an exhaustive search to identify the rotation angles around the mass center of the skull. The ideal midline would be the line passing through the mass center point with an optimal rotation angle, with respect to the original vertical direction of the CT image [68].

As deep learning models continue to evolve, their application to the diagnosis of TBIs is promising, particularly in enhancing the accuracy and efficiency of neuroimaging in the context of diagnosis. Potentially, these models can assist and further stratify the diagnosis of TBIs using protein biomarkers when neuroimaging alone is incomplete. Due to the nature of AI, large objective datasets are necessary for learning, which can be challenging for imaging purposes. For this reason, AI may be more impactful in assessing patient outcomes of TBIs and may also enhance the current clinical tools used in patient care.

As we have come to appreciate the impact of AI in improving the diagnosis of TBI, it can provide a positive preliminary analysis of patient conditions to inform how we treat them. AI can also inform patient care over the course of treatment. We must also investigate AI technology as a tool for predictive analysis. This can inform how we assess and treat patients based on anticipated clinical outcomes.

## 5. The Use of AI as a Predictor for TBI Health Outcomes

Machine learning algorithms have shown high potential as predictive models for estimating outcomes and mortality in TBI patients. This shows promise in how we proceed with clinical interventions and therapies in the rehabilitation of patients with TBI.

### 5.1. The Current Predictive Models: CRASH and IMPACT

There have been many studies into determining predictive factors of outcomes for TBI patients and strongly associated predictors have been identified such as GCS scores, age, and pupillary reaction [69,70]. Alone, these variables are not sufficient to provide a prognosis; however, when taken as a whole, these factors provide physicians with vital information and allow them to more precisely predict patient outcomes. Models like Corticosteroid Randomization After Significant Head (CRASH) and International Mission for Prognosis and Analysis of Clinical Trials in TBI (IMPACT) attempt to streamline this process by quantifiably predicting a patient’s outcome based on their measured predictive variables [71].

In a multicenter study assessing 10,008 patients with mild-to-severe cases of TBI within eight hours of injury, the CRASH model predicts death at 14 days, and death or disability at six months [72]. The IMPACT model predicts mortality and unfavorable outcomes (death, vegetative state, severe disability) at six months in a study on 9036 patients across three randomized trials and two observational series containing prospectively collected individual patient data on moderate-to-severe cases of TBI [73]. Both models utilize data from extremely large data sets that involve thousands of subjects each. Both models use multivariable logistic regression calculations to predict their outcomes, and both have an online version that allows clinicians to calculate clinical scores [72,74]. The basic CRASH model uses four predictor variables: age, GCS scores, pupil reactivity, and presence of major extracranial injury. The core IMPACT model uses three predictor variables: age, the motor score component from the GCS, and pupillary reactivity. In addition, the CRASH model has two different prediction models for high and low to middle-income countries [72,74].

While both models have undergone multiple validation studies, there is still contention between clinicians about the utility of the CRASH and IMPACT models. Most studies agree that the receiver operating characteristic area under the curve (AUC) values for these models range between about 0.6–0.9 [29,73,75,76,77], yet certain teams have found that the models are not as accurate as they may seem. Some sources indicate that the models may incorrectly prognose as much as one in four patients, especially when predicting outcomes beyond six months [77]. The discriminative validity of the models remained consistent over time and comparable to earlier recovery time points (AUC = 0.77–0.83). Both models had poor fit for severe outcomes, explaining less than one-quarter of the variation in clinical outcomes for cases of severe TBI [77].

The CRASH and IMPACT models, while not used as the main tools of prognosis, are useful prediction models that can help supplement a clinician’s decision-making. No prediction model can be 100% accurate, but the advancements in machine learning have created a new avenue of development for more accurate predictive algorithms based on artificial intelligence.

### 5.2. AI Predicting Functional Outcomes

Many of the experimental machine learning models calculate predictions similarly to CRASH and IMPACT. That is, they predict an outcome based on the values of predictor variables like GCS or demographic information. The majority of these studies are retrospective, and thus teams are able to compare the model’s estimation with the reported outcome or score of the discharged patients to assess its accuracy. While the CRASH and IMPACT models only predict mortality or unfavorable outcomes, machine learning algorithms attempt to make more specific predictions such as a patient’s functionality at discharge.

This was the basis of Say et al.’s study in 2022 when they performed the first known investigation of predictive statistical modeling of functional independence measure (FIM) scores for TBI patients [78]. The FIM score is a widely accepted functionality score based on 18 different areas of motor and cognitive actions developed by the American Academy of Physical Medicine and Rehabilitation and the American Congress of Rehabilitation Medicine [79,80]. The team tested eight different models based on either typical statistical methods or machine learning algorithms. Utilizing 40 different predictor variables like demographic information, comorbidities, and FIM score at admission, the team found that tree-based machine learning algorithms such as random forest (RF) and XGBoost were much more accurate than the standard statistical methods like parallel ordinal regression and predicted on average within ±1 from the true FIM scores of the patient at discharge for 14 of the 18 different categories [80]. The median reliability coefficients ranged from 0.61 to 0.90. The reliability coefficients for motor FIM scores were generally higher than the cognitive or communication FIM categories [80].

**Figure 2:**
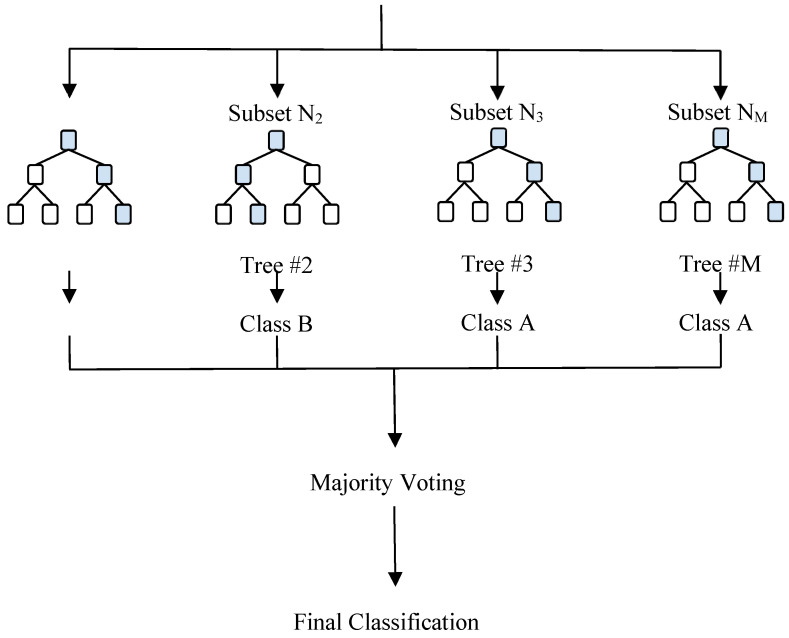
Sample random forest model. The random forest algorithm consists of these key steps: (1) For each tree in the forest, the algorithm randomly samples a set of training data. (2) The algorithm builds multiple decision trees using these samples, with each tree constructed independently using a random subset of features at each split. (3) A random subset of features is considered at each split. This technique, called feature bagging, creates diverse trees and reduces their correlation. In classification tasks, each tree votes for a class, The final classification for each set of data is decided by the majority of votes from decision trees of the predicted classes.

Satyadev et al. achieved similar results when exploring a machine learning algorithm that could predict discharge disposition after TBI [81]. Using data from 5292 patients treated for mild-to-moderate TBI at Duke University Hospital and 84 different predictor variables like vitals, demographics, mechanism of injury, and GCS score at admission, the team similarly compared the accuracy of standard statistical models such as logistic regression with machine learning ones like RF and XGBoost. Instead of predicting an outcome score like FIM, these models were tasked with estimating if the patient at discharge had a good, poor, or mortality outcome. A good outcome was determined as discharged to home or acute rehabilitation, a poor outcome included discharge to places such as nursing home care or long-term acute care, and a mortality outcome meant discharge to hospice or death. The team similarly found that machine learning models outperformed traditional statistical ones, including logistic regression, and that the RF model with an AUC of 0.84 (95% CI 0.81–0.87) was the most accurate with a successful prediction rate of around 83% [81]. Figure 2 below presents a sample random forest model, outlining its key steps, including random sampling of training data, independent construction of multiple decision trees, and feature bagging to enhance model diversity and reduce correlation. The final classification is determined by majority voting among the decision trees.

AI models have also been developed to predict mortality. One study exploring in-hospital mortality of TBI patients compared three different machine learning algorithms with multivariable logistic regression: artificial neural networks (ANN), support vector machines (SVM), and decision trees (DT) like RF. Again, machine learning algorithms outperformed linear regression, but SVM was actually found to be the most accurate, with an accuracy of 95.6% [82]. This has been contested; however, as other studies similarly exploring in-hospital mortality prediction found that RF models were more accurate than SVM [83].

These articles are representative of a plethora of experiments exploring the use of machine learning in predicting outcomes of TBI patients and provide a promising base for future research and refinement; however, not all of the present data shows such optimistic results. Zhang et al. found that their machine learning models, including RF and XGBoost, performed at levels comparable to, or worse than, conventional statistical methods like logistic regression when trying to predict the Barthel Index (BI) score of a TBI patient at discharge [84]. Christodoulou et al. performed a systematic review that explored 71 different studies that compared the predictive accuracy of machine learning algorithms versus logistic regression, and they found that overall, there was no evidence of AI models, like RF, being more accurate than standard logistic regression methods [85]. RF typically requires a larger dataset to build multiple decision trees effectively. In studies with limited data, RF may not be able to create diverse trees, leading to overfitting of data. Logistic regressions can still perform with limited data, especially when studying linear relationships [85].

While the studies included in this review did not only focus on predicting outcomes for TBI patients, clearly there is not a consensus that machine learning algorithms are better than the current conventional predictive methods. Our understanding of artificial intelligence has certainly grown, but there are still many limitations to the application of machine learning models in predicting TBI outcomes; thus, further research is needed before an AI model can be utilized regularly in the medical field.

There is a potential for predictive analysis when taking care of TBI patients. We are still far from consensus on how to implement standard conventions in this part of their care. Current clinical trials can provide better insights into how AI technology can enhance clinical decision-making.

## 6. Clinical Trials of AI in Traumatic Brain Injury

The integration of AI in clinical trials for TBI management has significantly advanced the field, offering new possibilities for diagnosis, prognosis, and treatment. One notable ongoing clinical trial investigated the use of AI-enhanced CT scans to monitor TBI. In this interventional study, 30 patients underwent CT scans at multiple time points (days 0, 1, and 3) and had intensive follow-ups, including daily assessments in the ICU during the first week, a follow-up at day 28 if hospitalized, and a final evaluation at six months via phone call to assess neurological outcomes. The primary aim was to utilize AI to differentiate tissue evolution post-TBI by analyzing scan profiles and correlating them with therapeutic intensity [86]. This approach sought to address the limitations of manual CT analysis, which lacks effective quantification of lesion evolution. By providing new quantitative insights, AI could significantly enhance the prognosis and treatment strategies for TBI patients, offering a more detailed and dynamic understanding of the injury’s progression and response to therapy. The final results of this clinical trial are pending [86].

Similarly, a 2023 survey from Rajaei et al. focuses on developing AI-based decision support systems for TBI, covering diagnosis, severity assessment, and long-term prognosis of complications. This study used a literature survey to highlight how AI can analyze large datasets from various diagnostic tools to provide a comprehensive assessment of a patient’s condition, helping clinicians make more informed decisions. The AI system’s ability to integrate data from imaging, clinical records, and patient history ensures a holistic approach to TBI management. The downstream effects include potentially reducing the risk of misdiagnosis with comprehensive analysis and informing patient care management for the clinical team [87].

Another pivotal study evaluated the effectiveness of the qER AI software in reducing turnaround times for CT head scans across four National Health Service hospitals in the United Kingdom. qER is an AI-powered tool that can analyze non-contrast CT scans of the brain. It can identify potential abnormalities and create a list for reporting scans based on clinical urgency. The qER device, a CE Class II approved software, detects and localizes six key abnormalities in non-contrast head CT scans, including intracranial hemorrhage, cranial fracture, midline shift, mass effect, atrophy, and hypodensities suggestive of infarcts [88,89]. This multi-center observational study of an estimated 16,800 patients used a stepped wedge cluster randomized trial that spanned 13 months and aimed to assess qER’s impact on reporting time, emergency pathway utility, safety, technical performance, and cost-effectiveness. The primary outcome focused on reducing turnaround times, while secondary outcomes included workflow support and economic analysis. By prioritizing scans with critical findings, qER aimed to streamline the radiology workflow, enhance diagnostic efficiency, and ultimately improve patient outcomes. The final results of this clinical trial are pending [88,89].

A 2021 review study from Wang et al. highlights the progress in integrating medicine and engineering for TBI rehabilitation, showcasing AI’s role alongside brain–computer interfaces, noninvasive brain stimulation, and wearable-assistive devices. Their research illustrates how AI can facilitate personalized rehabilitation programs by continuously monitoring patients’ progress and adjusting therapies in real time. This dynamic approach ensures that patients receive the most effective treatments based on their evolving needs, ultimately enhancing recovery outcomes [90].

**Figure 3:**
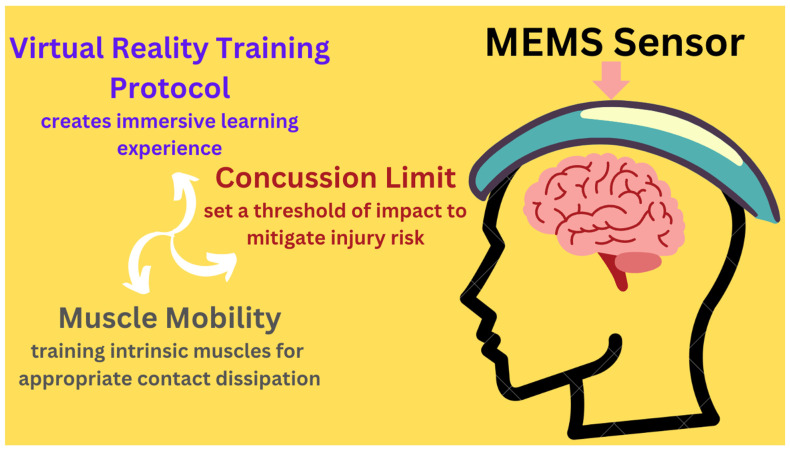
A MEMS sensor helmet. This figure shows the feedback mechanism that informs how AI technology is used to provide a comprehensive approach to concussion risk assessment and mitigation. A subject is taken through a simulated training session to experience similar movement patterns as in real life. The subject is fitted with a MEMS sensor which uses a machine learning algorithm to learn the strength and mobility of the head and neck of the user. Sensor feedback can provide data on how the intrinsic muscles of the head and neck are reacting to physical stimuli.

Another significant ongoing clinical trial is an interventional study of 100 people. It assessed the use of AI-enhanced Micro-Electro-Mechanical Systems (MEMS) sensors to screen and mitigate concussive risks in soccer players. MEMS sensors convert a range of physical stimuli, such as speed, acceleration, and rotation into an electrical signal [91]. This is achieved using small electrical chips that can interact with the environment and detect changes in a measured quantity [91]. The electrical signals provide real-time measurements that can be usable data for clinical monitoring. This study, structured as a randomized parallel assignment model with single participant masking, involved two key components: field-testing the sensors to establish personalized concussive thresholds and utilizing virtual reality (VR) training protocol to increase neck stiffness, thereby potentially reducing concussion risks [92]. Participants were randomized into trained and control groups, with smart sensors monitoring neck stiffness and head impacts throughout the trial. The primary goal was to optimize and finalize the sensor and training protocol, conducting field tests to validate their effectiveness in real-world settings. This trial aimed to enhance diagnostic accuracy and concussion prevention through personalized thresholds and conditioned responses, providing a comprehensive approach to concussion risk assessment and mitigation. The final results of this clinical trial are pending [92]. Figure 3 depicts a MEMS sensor helmet, illustrating its feedback mechanism in AI-driven concussion risk assessment and mitigation. The figure demonstrates how a machine learning algorithm analyzes head and neck strength and mobility during a simulated training session, using sensor feedback to assess muscle responses to physical stimuli.

A 2020 published clinical trial from Sheridan et al. comprised a prospective study of 73 trauma patients less than 15 years of age with suspected traumatic brain injury at a level 1 pediatric trauma center in Portland, Oregon. The study compared QuickBrain MRI to CT for pediatric head trauma detection to highlight advanced imaging techniques and possibly incorporate AI algorithms to improve TBI diagnostic accuracy. This study underscores the potential of AI to enhance imaging interpretation, reducing the time needed to diagnose and treat pediatric TBI. By providing quicker and more accurate diagnoses, AI-driven imaging techniques can significantly reduce the risk of long-term complications in children. QuickBrain MRI displayed a sensitivity of over 95% for the detection of clinically important TBI in pediatric head trauma [93].

In the realm of prognostic modeling, two 2022 studies from Eagle et al. and Ran & Azad highlight the challenges in accurately predicting TBI outcomes, stressing the need for caution when utilizing existing models for clinical decision-making. These studies emphasize the potential of AI to improve prognostic accuracy by incorporating a wider range of variables and learning from large datasets. However, they also caution that AI models must be continuously validated and updated to remain reliable [94,95].

In summary, these clinical trials (Table 1) underscore the transformative potential of AI in the management of TBI. By improving diagnostic accuracy, reducing turnaround times, and providing personalized risk assessments, AI technologies can significantly enhance patient care. Future research should continue to refine these technologies, validate their efficacy across diverse populations, and explore new applications to further advance the field of TBI management. The integration of AI into clinical practice holds promise for more precise, efficient, and effective care for patients suffering from traumatic brain injuries.

There are many promising ideas in clinical trials for AI applications in TBI patient care. In the long-term care of such patients, we must balance our desire for advanced technology with the principles of holistic medicine. There are many considerations for the long-term outlook of implementing AI into the care of TBI patients.

## 7. Long-Term Considerations for TBI Patients and AI-Assisted Treatments

TBI is a multifaceted disorder that necessitates a comprehensive approach to patient care over an extended period. Effective management involves not only addressing the immediate medical needs of the patient but also considering their overall lifestyle changes. This includes developing strategies to help patients adapt to any cognitive, emotional, or physical challenges they may face as a result of the injury. Furthermore, it is essential to integrate innovative care initiatives and rehabilitation programs that can enhance recovery and improve the quality of life for individuals living with TBI. Collaboration among healthcare professionals, patients, and their families is crucial to creating a holistic care plan that meets the unique needs of each patient.

### 7.1. Management of Patient Lifestyle

TBI patients face several challenges that can significantly impact their cognition, physical health, and psychological well-being. These challenges represent barriers to their care and personal agency in rehabilitation.

Cognitive dysfunction is a common outcome after TBI. There is a strong dose–response relationship between the severity of the TBI and the probability and/or extent of cognitive dysfunction [96]. Cognitive deficits are prevalent in the acute to chronic phases after TBI, with memory and attention deficits reaching 31% and 20% in the acute phase, 26% and 18% in the subacute phase, and 54% and 57% in the chronic phase after moderate-to-severe TBI, respectively [97]. TBI can lead to cognitive-communication disorders (CCDs). These disorders affect information processing and social communication skills and can have significant implications for a person’s quality of life, independence, and social participation [98]. These deficits can be addressed with the current INCOG 2.0 Guidelines for Cognitive Rehabilitation, which highlights best practices to promote the patient’s voluntary participation, stability of performance, and context-sensitive treatment [99].

Motor dysfunction can manifest in TBI patients as weakness, spasticity, and coordination problems [100]. This affects mobility and performing activities of daily living (ADLs) like dressing, bathing, and eating [101]. This creates a significant impact on patient autonomy and independence as they will require more assistance from caregivers. Depending on the severity of TBI, patients can be treated with an appropriate level of physical activity. It is important to note how these patients tolerate exercise over time. Appropriately managed exercise programs can offer far-reaching benefits to patients with TBI, positively impacting their overall well-being and quality of life [102,103]. Furthermore, early intensive physical rehabilitation management could be beneficial for neurologic function and activities of daily living in patients with moderate TBI [104].

Dealing with multiple limitations after a TBI can have a significant impact on a patient’s resting mood and outlook on life. Many patients experience depression, anxiety, and emotional lability, which can aggravate pre-existing challenges. Psychological well-being can be managed in TBI patients through different interventions. Cognitive-behavioral therapy (CBT) has been shown to reduce psychological distress and improve cognitive functioning among patients with mild and moderate TBI [105]. When Goal Management Training (GMT) is combined with external cueing and an emotional regulation module, this is associated with improved emotional regulation, psychological function, and quality of life in patients with chronic brain injury symptoms [106]. Addressing mental health challenges and improving overall well-being is crucial in ensuring patient engagement and outlook during rehabilitation.

### 7.2. Management of Caregiver Ethics

When managing patients who have experienced a TBI, it is essential to consider the role and needs of the caregiver. Caregivers often play a vital role in supporting the patient’s recovery and daily functioning. Understanding their challenges, providing them with the necessary resources, and ensuring they receive adequate support can significantly enhance the overall care of the patient [107]. Additionally, addressing the physical, emotional, and psychological demands placed on caregivers is crucial for maintaining their well-being and enabling them to provide effective care for the patient [108]. This makes the management of caregiver ethics in TBI patients an important consideration. This includes careful assessment of the risks and benefits of clinical interventions and rehabilitation therapies.

It is important to administer a neuropsychological assessment to determine the patient’s decision-making abilities in the event of a TBI. Studies have shown that medical decision-making capacity is mostly intact in patients with mild TBI, but is increasingly impaired in patients with complicated mild and moderate/severe TBI [109]. Caregivers are required to act in the best interest of the patient and understanding their patient’s level of decision-making capacity is important in discussing the long-term plan for their care. Specific interventions such as a decompressive craniectomy, a life-saving intervention for severe TBI, raise ethical issues regarding consent and resource allocation as they can result in severe neurocognitive impairment [110].

There is a significant commitment required of caregivers of individuals who have suffered TBI.

They often take on multiple responsibilities, including managing medical appointments, administering medications, and coordinating therapeutic interventions. In addition to the physical aspects of care, caregivers also offer emotional support, helping their loved ones cope with the challenges of TBI recovery, such as cognitive changes and mood fluctuations. Their unwavering dedication and understanding are vital in creating a nurturing environment that fosters healing and independence. This requirement may put caregivers of individuals with TBI at risk of serious burden, which can be influenced by the severity of the injury, functional disabilities of the patient, and unmet needs [111]. Interventions that target the unique needs of caregivers, such as educational, stress and anxiety self-management, coping, and emotional support components, have been shown to be beneficial [112]. Furthermore, caregivers may seek resources and support groups to navigate their own challenges, emphasizing the importance of self-care while caring for someone with TBI. Ensuring that the critical personnel involved in the care of individuals with TBI is paramount in giving them the best prognosis for rehabilitation.

### 7.3. Management of Ethics in the Implementation of Artificial Intelligence

The integration of AI into clinical care and decision-making needs careful ethical management to ensure patient safety, privacy, and equity. AI models require the highest quality and diverse datasets for training. Incomplete, biased, or unrepresentative datasets can lead to inaccurate or non-generalizable results [113]. Limited sharing of datasets that accurately represent disease and patient diversity can limit the generalizability of AI algorithms in healthcare [113].

The datasets that fuel AI technologies may not be representative of the population, leading to over- or under-representation of certain age groups, genders, races, ethnicities, abilities, or socioeconomic statuses. This could result in AI models that perform well on the training data but perform poorly in diverse real-world settings [114]. Also, there is often an inability to fully assess patient diversity used for algorithm development and testing, which can lead to biased outcomes [114]. This can lead to inaccurate healthcare outcomes and exacerbate present health disparities [114,115,116].

The development and implementation of biased AI systems can undermine the delivery of care, revealing inequalities in how patients are diagnosed, treated, and billed [117]. This may limit the effectiveness of AI-based solutions for many patients impacted by TBI. Cost and resource requirements can often be a prohibitive factor. The utility of AI for medical decision-making in global health and low-resource settings is hampered by limited diversity, transparency, and participation of resource-poor health institutions in AI technology production and validation [118].

There are different data points to consider in the course of care of TBI. This can include imaging, clinical assessments, rehabilitation targets, and any extenuating circumstances of the patient. Multimodal data integration is vital in TBI research as it builds a comprehensive understanding of the condition. It also aids in the development and implementation of predictive models and therapeutic interventions. The creation of common data elements (CDEs) for TBI research has been supported by multiple agencies to enhance consensus among researchers and facilitate the integration of diverse data sources [119,120]. The integration of different types of data points from separate sources is challenging but holds promise to yield larger and more robust samples that improve the potential significance and generalizability of research targets [121].

It is crucial to consider the public’s perspective and those it serves when incorporating AI into clinical care. The application of AI-based analyses to private medical data raises ethical concerns. While AI has the potential to advance clinical research, its use must be carefully regulated. Where AI is employed, care should be taken to openly acknowledge its application, credit the developers of the technology, and ensure that its use adheres to clear ethical guidelines and regulatory standards [122]. AI technologies often lack transparency in their algorithms and trained data. This creates difficulty in understanding for the general public on how they arrive at specific conclusions, which can thwart clinical trust and acceptance [123]. Balancing the promise of AI in healthcare with the protection of patient privacy and ethical considerations is crucial for responsible innovation in medical research. Overall, public opinion on AI in healthcare is mixed. There remains a strong preference for human medical professionals to make final decisions, despite the prevailing belief that they are more likely to make culturally biased decisions than AI [124]. Research should prioritize enhancing the effectiveness of clinical interventions and rehabilitation therapies.

### 7.4. Future Directions in AI and Emerging Technology Research for TBI Rehabilitation

The future is filled with exciting possibilities for the implementation of AI in the rehabilitation process for individuals with TBI. AI technologies have the potential to enhance various aspects of medical treatment in this field. AI technologies have the potential to enhance various aspects of medical treatment in this field. For instance, AI algorithms, particularly those based on machine learning, can analyze large imaging (e.g., CT, MRI) datasets to identify patterns and anomalies indicative of TBIs. These algorithms can improve the sensitivity and specificity of detecting pathologies and structural abnormalities compared to human radiologists [125].

AI has aided in swiftly and accurately classifying injury severity, which is essential for efficiently determining the appropriate level of care. AI can assist healthcare professionals in making more accurate diagnoses and treatment plans, leading to improved outcomes.

AI models can be trained to predict outcomes based on initial imaging. This helps clinicians to stratify patients and prioritize those at the highest risk and severity [125]. AI can detect subtle changes in brain structure and function over time, enhancing disease monitoring and treatment application [125]. AI can improve the personalization of rehabilitation programs for patients after TBI through various modalities. AI algorithms, such as artificial neural networks, have been used to predict in-hospital survival following TBI. This technology outperforms clinicians and traditional methods in terms of accuracy, sensitivity, specificity, and discrimination [126]. AI can integrate imaging data with pertinent clinical information to personalize rehabilitation plans. This includes tailoring rehabilitation programs and projecting a recovery timeline [125]. Moreover, AI tools can provide real-time feedback to patients, helping them to stay motivated and engaged in their recovery.

AI has demonstrated efficacy in cognitive rehabilitation. Leading to clinically meaningful improvement in numerous cognitive domains in people with TBI [127]. AI can provide detailed maps of affected brain regions, thus helping to minimize damage to critical areas during surgical procedures [125]. AI can also assist in the development of biomarkers to mark patterns and predictors of recovery outcomes. AI can also be used with blood biomarkers to improve diagnosis and prognosis following mild TBI [128].

Natural language processing technology develops advanced speech and language therapy tools that adapt to electronic health records (EHRs). AI has been leveraged to extract information from data in electronic EHRs. This could enable automated chart review for identifying distinct clinical characteristics [129].

Robotics and the use of AI-driven exoskeleton technology offer promising advancements in physical rehabilitation, providing adaptive support for motor function recovery [130]. Its application in conjunction with conventional physical therapy improves the patient’s recovery process. There should be priority given, however, to developing exoskeleton technology with ergonomics and portability in mind for increased ease of access [130].

Virtual reality combined with AI can create immersive, interactive environments for patients. These virtual environments create a “positive distraction” that increases patient engagement on the current rehabilitation tasks. This technology has been impactful in facilitating cognitive and motor rehabilitation interventions [131] and has also been helpful for post-traumatic stress disorder, which is often present in patients with TBI [132].

Neurofeedback systems integrated with AI can provide real-time monitoring of brain activity and biomarkers. The patients can be fitted with wearable sensors that provide this information remotely to caregivers [133]. This live feed of information can support clinical decision-making as well as patient engagement and compliance with rehabilitation plans [134].

AI is a game-changer for long-term healthcare. It can revolutionize remote monitoring and personalized rehabilitation plans for patients. AI can be instrumental in early detection, preventing complications before they arise. By capturing large datasets, AI could create tailored treatment plans that increase effectiveness and patient satisfaction. AI’s predictive ability enables proactive interventions, potentially reducing hospitalization rates and healthcare costs. Looking ahead to the future, it is not just about treatment—it is about transformation and making healthcare more accessible, efficient, and patient-centered. As we embrace emerging technology in the care of our patients, we must always prioritize humanistic values. It is paramount to balance the optimism in new ideas with the reality of living with a long-term condition for TBI patients.

## 8. Conclusions

AI has emerged as a pivotal tool in redefining TBI rehabilitation, bridging gaps in traditional care with innovative, data-driven approaches. While its potential to enhance diagnostic accuracy, outcome prediction, and individualized therapy is evident, challenges such as bias in datasets and ethical implications must be addressed. Continued research and multidisciplinary collaboration will be key to harnessing AI’s full potential, ensuring equitable access and optimizing recovery outcomes for TBI patients.

Overall, the integration of AI in TBI rehabilitation presents numerous opportunities to advance patient care and enhance the effectiveness of therapeutic interventions.

## Figures and Tables

**Table 1:** Key studies on the application of AI in TBI research and clinical practice.

Study	Year	Focus	Key Findings/Objectives	Status
**Clinical Trial (University Hospital, Grenoble)**	2021-present	Use of AI to analyze CT scans for TBI progression	Aims to differentiate brain tissue evolution post-TBI and correlate with therapeutic intensity; addresses limitations of manual CT analysis	Pending results
**Review Study [87]**	2023	AI-based decision support systems for TBI	Highlights comprehensive assessment tools for diagnosis, severity assessment, and long-term prognosis; integrates data from various diagnostic tools	Completed
**Clinical Trial (UK NHS Hospital Registry)**	2024-present	Effectiveness of qER in reducing CT head scan turnaround times	Multi-center trial across 4 NHS hospitals in UK; assesses impact on reporting time, emergency pathway utility, safety, and cost-effectiveness	Pending results
**Review Study [90]**	2021	AI in TBI rehabilitation	Showcases AI’s role alongside brain-computer interfaces and wearable devices; facilitates personalized rehabilitation programs	Completed
**Clinical Trial (AI-enhanced MEMS Sensors)**	2021-present	Use of AI-enhanced sensors to screen and mitigate concussive risks in soccer players	Establishes personalized concussive thresholds; uses VR training to assess neck stiffness	Pending results
**Clinical Trial (QuickBrain MRI)**	2017–2019	Comparison of QuickBrain MRI to CT for pediatric head trauma patients	QuickBrain MRI showed >95% sensitivity for detecting clinically important TBI in pediatric head trauma	Completed
